# Personalized Privacy Assistant: Identity Construction and Privacy in the Internet of Things

**DOI:** 10.3390/e25050717

**Published:** 2023-04-26

**Authors:** Kai-Chih Chang, Suzanne Barber

**Affiliations:** Department of Electrical and Computer Engineering, The University of Texas at Austin, Austin, TX 78712, USA

**Keywords:** identity, privacy, Internet of Things

## Abstract

Over time, the many different ways in which we collect and use data have become more complex as we communicate and interact with an ever-increasing variety of modern technologies. Although people often say they care about their privacy, they do not have a deep understanding of what devices around them are collecting their identity information, what identity information is being collected, and how that collected data will affect them. This research is dedicated to developing a personalized privacy assistant to help users regain control, understand their own identity management, and process and simplify the large amount of information from the Internet of Things (IoT). This research constructs an empirical study to obtain the comprehensive list of identity attributes that are being collected by IoT devices. We build a statistical model to simulate the identity theft and to help calculate the privacy risk score based on the identity attributes collected by IoT devices. We discuss how well each feature of our Personal Privacy Assistant (PPA) works and compare the PPA and related work to a list of fundamental features for privacy protection.

## 1. Introduction

Identity privacy and the protection of Personally Identifiable Information (PII) is about giving people meaningful choices when collecting, using, and sharing data, and providing them with enough detailed information about those choices to make informed decisions. Very few people read privacy policies carefully and exercise some of the powers at their disposal when engaging in online activities. Users often read a small part of a website’s privacy policy if they read the privacy policy at all. Compounding the problem, it is often difficult for users to quickly and clearly know what information will be collected, with whom and how their data is share, and the consequences if their data is abused or breached. Meanwhile, everyday objects become internet-connected and, the Internet of Things (IoT) has progressively become pervasive in our lives.

However, users often do not notice IoT devices nearby at all, not to mention the data collected and shared by these devices and the downstream consequences of data abuse and breach. As the IoT continues to grow and evolve, concerns around personal privacy remain a major issue. A 2023 survey by Deloitte found that 50% of consumers were concerned about the security of their personal information when using IoT devices, while 41% worried about their data being shared with third parties [1]. This is not without reason, as IoT devices collect vast amounts of data on users’ behaviors and preferences. In addition, it was reported that the average cost of a data breach in the United States is $9.05 million [2] and it was also reported that over 61 million records have been compromised in a data breach involving IoT devices [3]. This breach included sensitive information such as names, birth dates, and Social Security numbers, leaving affected individuals vulnerable to identity theft and other forms of financial fraud.

These statistics demonstrate the urgent need for manufacturers and policymakers to prioritize IoT security and privacy measures. We need a standard mechanism for discovering these IoT devices, detecting the data they collect and share, let alone informing users about the options they could choose and the consequences of the action they choose. There is a widespread disregard or desperation for reseizing the collection and usage of identity attributes, or Personally Identifiable Information (PII), as this requires a novel and extensible design that empowers users with the capability of controlling and understanding of their own identity attributes.

This research is working on IoT Personalized Privacy Assistants (PPAs) to help notify users of the data collection, sharing and use practices of nearby IoT devices to help users better understand the consequences (the potential monetary loss) of such IoT data practices, making them more likely to make appropriate decisions if options are given. To achieve this goal, this research utilizes a privacy risk score framework, which is capable of quantifying a user’s privacy risk when connecting to an IoT device. Specifically the proposed PPA calculated the risk associated with exposing personal identity data, referred to as identity attributes in this research. The PPA can show users the set of identity attributes collected by the nearby IoT devices and calculate the privacy risk score using the UTCID Identity Ecosystem, which is a graph-based model describing identity attributes as nodes and the relationships between the identity attribute nodes as edges. This research will introduce each PPA feature and step-by-step calculation of privacy risks introduced by respective IoT devices.

The following are the primary contributions of this paper:

- **Personalized Privacy Risk Estimation**: User privacy risk calculations customized based on knowledge of user-specific identity attributes.
- **Personalized Privacy Assistant for IoT**: Mobile application that gives privacy risk notifications and recommendation actions for users when an IoT device collects or seeks to connect to their phone and collect the user’s identity attributes.
- **Informs users of consequences resulting from IoT Device collection & sharing of identity attributes**: Uses knowledge of the IoT Identity Ecosystem to evaluate the consequences of privacy vulnerabilities of mobile apps (sensitive identity attributes collected and shared by IoT).

The remainder of this paper is organised as follows: Section 2 briefly presents the background technologies needed. Section 3 provides references to the related literature while discussing the existing solutions. Section 4 presents the proposed solution while covering a wide range of problem statements and system executions. Section 5 details experimental results for PPA features while providing comparisons between existing work and some pointers for future work. Finally, Section 6 draws the conclusions.

## 2. Background

This section introduces the essential technologies leveraged by the proposed IoT Personalized Privacy Assistants (PPA).

### 2.1. Center for Identity

The Center for Identity at the University of Texas at Austin (UTCID) [4] is a research and education center that is focused on protecting individual privacy and identity in today’s digital world. The center offers a range of programs and services that help to develop and promote effective strategies for safeguarding digital identity. These programs include research initiatives, educational programs, and community outreach efforts. The Center for Identity also collaborates with industry partners, government agencies, and academic institutions to identify emerging threats and develop effective countermeasures. The center’s mission is to empower individuals and organizations to protect their digital identities and privacy, and to help build a more secure and trustworthy digital ecosystem. Overall, the Center for Identity is an essential resource for anyone who is concerned about protecting their digital identity and privacy.

### 2.2. Itap

The Identity Threat Assessment and Prediction (ITAP) [5,6] research project at the Center for Identity at the University of Texas at Austin models and analyzes identity theft and fraud cases including the vulnerabilities that make data compromises and breaches possible and the consequences of those data exposures. UTCID ITAP identifies the types of identity attributes exposed, the frequency of identity attributes are exposed and the consequences of those exposures-financial, reputational and emotional distress for victims.

Between years 2000 and 2023, about 6000 incidents have been modeled [7]. There are two methods by which ITAP collects identity theft details. Among them, these details include the identity assets exposed, the location and date of the incident, the age of the victim, and the method of the perpetrator. The first way is to monitor websites for cases of identity theft, fraud and abuse. These websites provide many reports on these cases, and many kinds of details required by ITAP will be included. The second way is to use Google Alerts. So ITAP researchers are notified instantly when new reports of identity theft, fraud and abuse emerge. By analyzing these cases, ITAP has generated a list of identity attributes reported in the identity theft, fraud and abuse cases with each of identity attribute being characterized according to a set of characteristics such as monetized value, risk (probability) of exposure, verification accuracy, and other characteristics depending on this empirical data. To date, ITAP has identified over 600 identity attributes involved in these theft and fraud cases, which is the list of identity attributes serving as the starting reference data for this research. This starting set of identity attributes is primarily used to describe People (e.g., ID numbers, mailing address, email address, mother’s maiden name, phone number, etc.).

### 2.3. Identity Ecosystem

In order to construct a comprehensive graph composed of identity attributes and the relationships between identity attributes in the UTCID Identity Ecosystem, this research set out to determine the identity attributes both found in the ITAP cases and collected or generated by IoT by conducting empirical studies related to IoT devices, mobile apps, and device services, and also referring to daily news and use cases [8].

### 2.4. Iot Privacy Architecture

This research enables privacy risk analysis of data collection by IoT devices and service. Therefore, we need a privacy infrastructure. Such a facility would allow IoT devices and resources to register and provide their location and identity information collected. By connecting the user’s mobile phone to this infrastructure, the user can know which IoT devices or IoT resources are nearby that may collect their identity attributes. Through the user’s GPS location, this privacy infrastructure can know the IoT devices near the user’s physical location. These include devices such as smart houses and smart cameras, services such as location tracking systems, body temperature analysis videos, and various smart applications. Taking services as an example, this infrastructure should allow users to choose whether to use it, such as whether to allow this device to track their physical location, but services such as body temperature detection at security checkpoints will not be available to provide users any options. Researchers have performed studies for IoT privacy architecture [9,10,11] and as a result, we are proposing a solution that utilize those studies. The proposed PPA in this research will be connecting to those privacy infrastructures, analyzing the information provided by those privacy infrastructures, and hence empowering users with abilities to control over their identity privacy in the Internet of Things.

## 3. Related Work

### 3.1. Identity Attributes in General

Identity attribute can be used on its own or can be combined with other information to re-identify an individual and hence causing irreversible consequences. Previous work has focused on how it is possible to detect any identity leakages and protect identity attribute from being exposed and shared. Online Social Networks (OSN) is a popular area. There was a study [12] focused on the OSN. OSN usually contains a great amount of users’ identity attribute. The research found out that identity leakages can occur by combining HTTP header information and cookies together. Twitter, as a great platform, contains an ocean amount of data that could be studied. A content analysis [13] mapped the amount and the type of PII included in the public Twitter messages and posts.

Aside from studies for networks, some novel tools have been invented. A tool [14] is developed to detect the hidden or unintentionally inserted identity attributes in online documents. Such documents could be manipulated by fraudsters to identify authors of the document and their organization. Another tool [15,16] focused on hidden or unintentionally inserted identity attributes from identity providers of federated identity management systems. Arefi et al. [17] proposed PIITracker which is capable of detecting if there is a transfer of a set identity attributes over the network.

### 3.2. Devices and Privacy

Related work addressing devices and personal privacy can be divided into several categories.

First, a great body of research is dedicated to the possibility of mobile apps accessing identity attributes. Canbay et al. [18] used J48 classification algorithm to detect if there is leakages of identity attributes from mobile applications and used machine learning to analyze mobile applications to distinguish if they are malicious or not. Reaedon et al. [19] performed leakage detection of mobile applications transferring identity attributes that those applications did not request permissions to access by monitoring mobile applications’ behaviour and network traffic. Liu et al. [20] performed static and dynamic analysis and showed that different sets of identity attributes are collected and leaked by the analytics libraries in popular Android apps. Alazab et al. [21] studied permission requests and API calls invoked by mobile applications and found that dangerous permissions are often used by mobile applications to access user’s identity attribute.

Second, researchers have also studied the transmission of personal data from mobile devices to any third parties. Grundy et al. [22] gathered more than 800 popular medicines related apps on Google Play to monitor the transmission of identity attributes to third parties. They made some suggestions which are just in line with the proposed concept of this research. Another research also was interested in health related mobile applications. Huckvale et al. [23] constructed a contemporary assessment for privacy policies of popular mobile apps and compared privacy policies to those mobile apps’ actual behavior to see if there is any data transmission to third parties. He et al. [24] found that most third-party libraries in popular apps that accessed users’ identity attributes has network connections and that there are 3 different types of identity attribute leakage paths in mobile apps. Shipp and Blasco [25] investigated privacy polices of popular Android menstruapps and found out that identity attributes like email address and account information are often well-covered in the privacy policy, but reproductive-related data is not and are often disregarded. This means that users’ reproductive cycle and sex life information could be access by fraudsters. Besides the apps on the devices, another important factor connecting these IoT devices is the Internet. The Domain Name System (DNS) protocol is the basis for the operation of the Internet. Using DNS to attack organizations and exploit server-side user data is also an increasingly common method. Using various open source tools, Salat et al. [26] provided various ways to detect DNS attacks and provided the most efficient strategies to deal with and detect these DNS attacks. When it comes to network, we have to mention the blockchain which has been widely discussed in recent years. With its decentralization and other properties, Rossetto et al. [27] produced a privacy architecture to store health-related data, and demonstrated the efficiency and security of their system.

Third, to protect user privacy, researchers have begun to explore techniques and analysis for mitigating digital privacy risk. Babun et al. [28] built the IoTWatcH which can notify users’ about the privacy risk of the IoT app by processing and analyzing the data transmission by utilizing the Natural Language Processing (NLP) techniques. Sharma et al. [29] performed a privacy risk analysis in Android applications with reference to machine learning technique and was able to identify permissions from malware application. Han et al. [30] discovered that the paid version of mobile applications are not really safer then their free version. Many of them reuse the the third-party libraries that they used in their free version. Pereira et al. [31] have created a tool that analyzes websites and generates reports to inform users of recommendations for complying with the regulations released by the General Data Protection Regulation (GDPR). Bluetooth Low Energy (BLE) is widely required by IoT devices, but due to its simple design and architecture, different vulnerabilities and risks also exist. Different types of vulnerabilities may have different attack scenarios. Barua et al. [32] analyzed them and list possible countermeasures and also provided examples of different vulnerabilities in IoT devices that actually use BLE.

### 3.3. Privacy Risk Estimation

This research divides the research in privacy risk into three categories: mobile App’s permission analysis, mobile App’s privacy policy analysis, and mobile security and privacy framework.

Several works have indicated that mobile applications are requesting a bunch of unnecessary permissions of users’ identity attributes. For example, a survey [33] was conducted to discuss the problem of over-declared permissions of operating systems for different type of popular mobile phone. A tool [34] for Android API was built to address the problem of over-declaration too.

There are a bunch of existing work talking about mitigating digital privacy risks. Zaeem et al. [35,36,37] proposed a technique that parses privacy policies and automatically generates summaries. A set of metrics [38] was built by constructing a list of questions and answering those questions by reviewing privacy policies. Harkous et al. [39] proposed a framework called Polisis based on neural-network classifiers which can perform analysis on privacy policies.

Indeed, breaches of identity attributes can lead to financial and emotional damage to users. A tool [40] was designed to help understand the origin of such scale of identity breaches and to identify the potential vulnerabilities from mobile apps based on how cloud APIs are used.

### 3.4. Privacy Assistant

Users of the IoT are often interacting with technologies they are not familiar with without realizing it. A lack of awareness and a lack of ways to effectively manage the multitude of notifications and settings makes it difficult for users of IoT resources. IoT users are often unaware of what devices or services around them are collecting their data, and what happens to them after it is collected.

Therefore, different kind of privacy assistant that remedy above situation and requirements has been invented. Das et al. [11] developed a privacy assistant that is able to memorize user’s privacy expectations and preferences with machine learning techniques. Their privacy assistant can distinguish whether the identity attribute collection is worth telling their users and only tells information that their users really care. Feng et al. [9] built a framework to help developers design meaningful privacy choices in their system or application. They also showed how their could be utilized for IoT privacy assistant. Ayci et al. [41] built a privacy assistant that leveraged deep learning. Their privacy assistant can adjust its recommendation based on user’s preference and can determine if it is certain about the recommendation and if not, it asks their user and this is how their personal assistant can personalize its recommendations. Stöver et al. [42] conducted a series of user studies to ascertain their vision of the PPA, in order to analyze how to design the PPA. For example, what functions should PPA have and which group of users are suitable for different functions.

### 3.5. Summary

Table 1 presents a summary of related work for identity attributes, personal privacy, IoT devices, and privacy assistants.

While significant work related to PII protection and detection has been reported, none have focused on the analysis both the range of identity attributes, identity attributes collected and generated by IoT devices and the relationships between identity attributes. Some research did recognize a short list of identity attributes in their work, it is still not enough to fully describe or identify an individual and associated security and privacy risks in the current complicated IoT society.

Existing work analyzes one mobile app at a time to detect identity attributes shared from or generated by the mobile app. The gaps are that it lacks comprehensive analysis of identity attribute collection, sharing and abuse (multiple cases/incidents of use and abuse) and that it lacks comprehensive analysis of identity attribute occurrences, dependencies, value and risk across multiple cases of sharing and abuse. This research builds a comprehensive model of identity attributes, attribute dependencies, attribute values and attributes risks based on empirical data across many mobile apps (experiments evaluating over 200 apps to date).

The gaps of existing work are that detection of actual data transmission only allows for reactive solutions and that consequences of personal data transmissions to third parties not identified. This research offers preventive solutions by evaluating privacy vulnerabilities in advance and informs users of consequences resulting from mobile app collection and sharing of identity attributes.

Nevertheless, the aforementioned personal assistants focus on users’ privacy and setting preferences and automate the setting and determination process with machine learning or deep learning techniques. On the other hand, this research builds the personal privacy assistant that is capable of telling the user the consequences of sharing identity attributes or allowing data collection for IoT devices and services.

## 4. Proposed Solution

**Figure 1:**
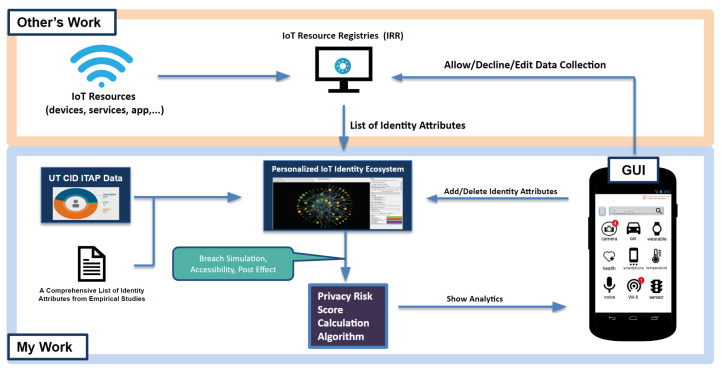
A diagram that shows the overview of this research.

In this section, we describe how we build the entire personalized privacy assistant step-by-step. In order to better follow what this research is introducing, Figure 1 shows the simple flow of this research. Looking from the most left of the diagram, this research first performs an empirical study to extract a list of identity attributes that are being collected by privacy policies, mobile apps, and IoT services. Then, with the reference to the Identity Threat Assessment and Prediction (ITAP), this research generates a dataset of identity attributes that could be taken as an input to the UTCID IoT Identity Ecosystem. The UTCID IoT Identity Ecosystem leaverages the Bayesian Network to build a graphical model with each node as an identity attribute and each edge as the relationship between identity attributes. Next, given a set of identity attributes that is collected by an IoT resource, the privacy risk score calculation algorithm generates the privacy risk score of that data collection based on the simulation results from the UTCID IoT Identity Ecosystem and hence shows the score in the graphic user interface (GUI) of the personalized privacy assistant. We will briefly introduce each step in the remaining of this section.

### 4.1. Find the Comprehensive List of Identity Attributes for People and Devices

To build a universal identity map with nodes and edges, we tend to find the list of nodes first. The privacy policy plays an important role.

Privacy policies help users understand what portion of their identity attributes would be collected and used or shared by a specific mobile application or website. A good privacy policy needs to have a list to tell users which identity attribute will be collected, and also tell users what those identity attributes are. Furthermore, the privacy policy has to tell users where they can find these collected identity attributes and why they should be collected, and at the same time inform users how these data are collected. Another thing that users need to pay attention to is whether this privacy policy tells users which people or organizations can see the collected data of users. This research manually processed the privacy policies of more than 200 mobile applications to see what set of information the apps collect. Then, this research mapped the information against the existing identity data compromised in the ITAP threat cases.

We used a structured review process to guide the collection of apps. The apps that have more than 300 thousand downloads and Privacy Policy are selected. The two biggest app stores are the Apple iOS App Store and Google Play store. The research selected the top 100 applications in the 8 most popular categories [43,44] (by the number of downloads) in the Apple App Store for iOS and in the Google Play Store for Android. These 200 apps handle sensitive user data across different categories: Games, Health, Business, Utilities, etc. As a result, we made a global list of identity attributes of people and devices.

### 4.2. Iot Identity Ecosystem

This research has set out to identify identity attributes collected or generated by IoT by conducting empirical studies related to IoT devices, mobile apps, and device services, and also referring to daily news and use cases. With the far-reaching list of identity attributes, this research builds the IoT Identity Ecosystem.

The UTCID IoT Identity Ecosystem organizes identity attributes describing People (e.g., blood type, postal address, name) and Devices (model number, name, GPS location). Of course some attributes might be used to describe both a Person and a Device (e.g., GPS location).

Recently the concept of “Smart City” has rapidly risen [45]. Smart Cities consists of smart phones, mobile devices, sensors, embedded systems, smart environments, smart meters, and instrumentation sustaining the intelligence of cities [46]. As a result, the relationship between people and devices has become more intertwined. From mobile phones and laptops to GPS, sports watches and even to baby monitors, technical devices are collecting identity attributes anytime and anywhere. This research constructed a list of identity attributes of items according to devices’ characteristic, function, affordances and other documents [47].

The UTCID IoT Identity Ecosystem takes UTCID ITAP dataset as input. Each identity attribute in this UTCID ITAP dataset has several characteristics such as “Attribute Type”, “Risk”, “Liability Value”, “Possession”, “Verification Accuracy”, “Prevalence”, “Uniqueness”, “Verification Invasiveness”, and “Probability of Exposure”. This research utilizes some of these characteristics in the proposed solution. For example, identity attribute’s type is divided into four categories: What You Are, What You Have, What You Know, and What You Do [48], while prior work focused on identity attribute type as relates to the people, this research extends the concepts and properties to identity attributes not only for people but also for devices.

- **What You Are**: For a person, it means a person’s physical characteristics, such as fingerprints and retinas. For a device, it means the type of a device. It can be a laptop, a smart watch, a sensor, and so on. It is also related to a device’s hardware configuration, such as circuit design and power usage.
- **What You Have**: For a person, it means credentials and numbers assigned to the person by other entities. For a device, it means identifiers assigned to the device by other entities such as model numbers, serial numbers, and inventory tags.
- **What You Know**: For a person, it means information known privately to the person, such as passwords. For a device, it means any information that is stored on the device and know only to the device.
- **What You Do**: For a person, it means a person’s behavior and action patterns, such as GPS location. For a device, it is related to application types, GPS location, behavior patterns.

**Figure 2:**
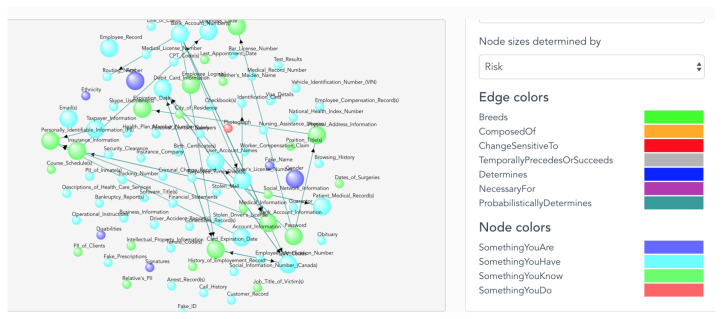
A snapshot of the IoT Identity Ecosystem. In this particular example, by utilizing the filter on the right-hand side, the size of the node is determined by the risk of exposure and different colors are used to distinguish the types of identity attributes. The name of each identity attribute has shown on each node.

With experts knowledge and studies, the research populated identity attributes of devices, classified those attributes and defined relationships between those device identity attributes to the identity attributes of people defined in previous work (see Figure 2). Table 2 shows examples of identity attributes of IoT devices. We will discuss more details of UTCID IoT Identity Ecosystem in the next subsection.

Accessibility and Post Effect

With the Bayesian inference, the UTCID IoT Identity Ecosystem answers several research questions relevant to the privacy risk, risk of exposure and liability of any person in terms of managing identity attributes. The graphic model in the IoT identity ecosystem does not represent a specific person. The graphic model in the IoT identity ecosystem shows a more general analysis for the universal relationships of identity attributes and miscellaneous risks for identity management. The question that IoT Identity Ecosystem can answer and also the functionality that this research utilizes is “When a set of attributes is exposed, how does it affect the risk of other attributes being exposed?”.

For instance, if the credit card number of an individual is compromised, what are the most risky node items that fraudsters might proceed to obtain after that? Moreover, what if one’s unique device identifier number is compromised? What are other set of device identity attributes that fraudsters might try to obtain in order to identify a specific person? Multiple attributes can be selected as evidence (i.e., exposed identity attribute) at the same time. It also shows potential loss after such a breach. The UTCID IoT Identity Ecosystem also allows the users to choose a node property, such as value or risk, to determine node sizes and colors in the 3D graphic model.

Let us define some notations before introducing more details. Given *V* as a set consisting of *N* identity attributes and *E* as a set of directed edges, the IoT identity ecosystem is represented as a graph G(V,E). Each edge is a tuple $e_{ij}=<i,j>$ where the identity attribute $A_{i}$ is the starting node and the identity attribute $A_{j}$ is the destination node such that 1≤i,j≤N. Each node represents an identity attribute that consists of different quantitative properties and each edge represents a possible path by which the destination node can be breached given that the starting node is breached. We assume there is no cycle in the graph of UTCID IoT Identity Ecosystem.

This research constructs two different approaches to calculate the impact on the identity attributes from their ancestors and descendants. The first one is a static approach.

Static Approach

This research has defined an identity attribute linked to a person or device. Among several different properties for attributes the research defined Risk and Uniqueness as follows:

- Risk (shows the risk of exposure): Low, Medium, High.
- Uniqueness (shows how unique the identity attribute is for the individuals, devices or organization who have it): Individual, Small Group, Large Group.

These properties are obtained from UTCID ITAP dataset. The Uniqueness of an identity attribute determines the strength of the identity attribute [49]. At first, by referring to the Bayesian Network Model in UTCID IoT Identity Ecosystem, this research endeavours to provide a approach that utilizes the basic properties of each identity attribute.

For example, the identity attribute “Social Security Number” has risk of “High” and has uniqueness of “Individual”. However, using this approach as a metric is not leveraging the characteristics of the Bayesian Network model. So we move forward to an advanced approach.

Dynamic Approach

For the advanced approach, or dynamic approach, this research uses Bayesian inference. Each identity attribute *A* has a prior probability, or probability of exposure, denoted as P(A) which indicates the probability of this identity attribute gets exposed on its own. Each identity attribute *A* also has a liability value denoted as L(A) which indicates the max amount of potential monetary loss one would encounter when the identity attribute *A* is exposed. Note that the probability of exposure and the liability value of identity attributes are obtained from UTCID ITAP dataset.

The first part of this dynamic approach is bringing the ancestors of an identity attribute into the calculation.

**Figure 3:**
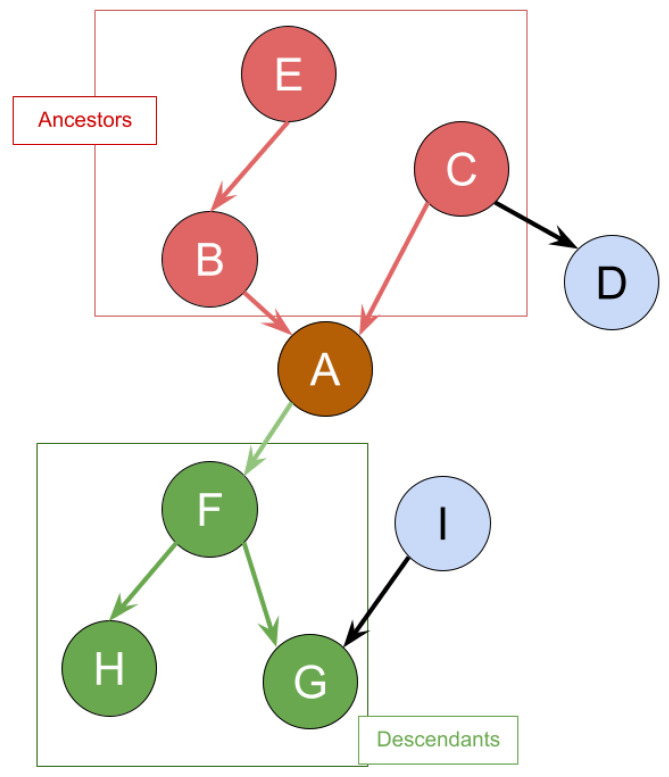
An example of ancestors and descendants.

Given the graph in Figure 3 as an example of IoT identity ecosystem, identity attribute B,C, and *E* are ancestors of identity attribute *A*, whereas identity attribute *D* is not the ancestor of *A*. Every ancestor of identity attribute *A* has a path that can lead to itself. Let Anc(A) be the set of ancestors of *A*. Given that $A_{k}$ is exposed where $A_{k}\in Anc\left(A\right)$, by applying the Bayesian inference, we can get the posterior probability of exposure of *A* which is denoted as $P^{\prime }\left(A\right)$. This probability indicates the probability of identity attribute *A* gets exposed on its own after its ancestor $A_{k}$ was exposed. For simplicity, we denote the posterior probabilities caused by $A_{k}$ as $P^{\prime }{\left(A\right)}_{A_{k}}$.

Hence, given the $P^{\prime }{\left(A\right)}_{A_{k}}$ values, it is easy to compute the percentage increase in the probability of exposure for identity attribute *A* as $\left(P^{\prime }{\left(A\right)}_{A_{k}}-P\left(A\right)\right)$. Therefore, the sum of the percentage increase in the probability of exposure for *A* can be computed as

(1) $$AC\left(A\right)=\sum_{A_{k}}\left(P^{\prime }{\left(A\right)}_{A_{k}}-P\left(A\right)\right)$$

where $A_{k}\in Anc\left(A\right)$ and we call AC(A) the “Accessibility” of *A*.

This research calls accessibility a dynamic property because it changes its value based on different situation (different set of identity attributes got breached). The value of accessibility of identity attribute *A* would be different if different ancestors of *A* is breached. The value of accessibility indicates the difficulty to get this identity attribute. The lower the value is, the harder to get to this identity attribute. The value of the accessibility could be affected by the size of the ancestors. It makes it harder to get to this identity attribute due to only few entrances.

The second dynamic property is called the “Post Effect”. Take the graph in Figure 3 for example again, identity attributes F,G, and *H* are descendants for identity attribute *A*. As a result, given that identity attribute *A* is breached, by applying Bayesian inference, the probability of exposure of every descendant of identity attribute *A* is going to be impacted. Let Des(A) be the set of descendants for identity attribute *A* and identity attribute in Des(A) be $A_{k}$. Hence, the posterior probability of exposure for identity attribute $A_{k}$ is $(P^{\prime }\left(A_{k}\right)$ and the percentage of the probability difference can be shown as $\left(P^{\prime }\left(A_{k}\right)-P\left(A_{k}\right)\right)$. Recall the each identity attribute *A* has a liability value L(A). Therefore, the increase for monetary loss for identity attribute $A_{k}$ can be denoted as $L\left(A_{k}\right)\cdot \left(P^{\prime }\left(A_{k}\right)-P\left(A_{k}\right)\right)$.

Thus, the total monetary loss increase of the descendants set Des(A) can be showed as

(2) $$PE\left(A\right)=\sum_{A_{k}}L\left(A_{k}\right)\cdot \left(P^{\prime }\left(A_{k}\right)-P\left(A_{k}\right)\right)$$

where $A_{k}\in Des\left(A\right)$ and we call PE(A) the “Post Effect” of *A*.

The post effect also changes its value based on different situation. In real life cases, fraudsters would not only target on one identity attribute of the victim. Surely multiple identity attributes will suffer. Therefore, as the identity attributes of different sets are stolen, the exposure probability of each identity attribute will also change, causing the Post effect to change accordingly. The higher the value of post effect for identity attribute *A* is, the larger the impact to the descendants of *A* will be.

Take “Social Security Number” for instance again. It has accessibility of 58% and it has post effect close to $14 million. Hence, with the two dynamic properties of the identity attributes, we are able to perform the privacy risk analysis in the next part.

### 4.3. Privacy Risk Score Estimation

To perform the privacy risk score analysis, we have to discuss what privacy risk is. This research defines the privacy risk as the danger of monetary loss one could have faced when their identity attributes are compromised. Thus, the privacy risk score refers to the level of risk a person is at. A higher privacy risk score indicates the financial loss that a person may face if their identity attributes are exposed to the wrong hands.

At first, this research utilizes *Risk* and *Value* for measuring the risk score of each identity attribute.

#### 4.3.1. Basic Measurement

The identity attribute in ITAP is assigned a value to represent the property damage that may be caused after the identity attribute is exposed, but this value is actually obtained by averaging the data obtained from more than 6000 identity theft, abuse, and fraud cases. To know the loss that exposure of an identity attribute may cause to the user should be viewed with the expected value. Here, we call it expected loss.

Given an identity attributes *A* in UTCID ITAP dataset, it has a monetary value L(A) and a probability of exposure P(A). This research first defines the expected loss of an identity attribute *A* as

(3) Exp(A)=P(A)·L(A)

Such static analysis only provides limited information and that is why this research proceeds further by leveraging two of the identity attribute’s dynamic properties.

#### 4.3.2. Dynamic Measurement

The two dynamic properties are just related to the probability of exposure and property damage, respectively. The *Accessibility* (defined as part of this research effort) is the influence of the ancestors of an identity attribute on its exposure probability, so we can superimpose this effect on the original exposure probability of the identity attribute. On the other hand, the *Post Effect* is an increase in the property loss caused by an identity attribute to its descendants, so we can also superimpose it on the original property loss of the identity attribute.

This research defines the new Risk (real risk) of exposure of an identity attribute *A* as

(4) $$P^{\prime }\left(A\right)=P\left(A\right)+AC\left(A\right)$$

where AC(A) denotes the accessibility of *A*.

This research defines the new value (real value) of an identity attribute *A* as

(5) $$L^{\prime }\left(A\right)=L\left(A\right)+PE\left(A\right)$$

where PE(A) denotes the post effect of *A*.

Hence, for dynamic measurement, we define the expected loss of identity attribute *A* as

(6) $$Exp\left(A\right)=P^{\prime }\left(A\right)\cdot L^{\prime }\left(A\right)$$

Since the range of the expected loss in UTCID ITAP dataset is from 0 to $10^{7}$. This range is a bit too broad for data analysis and comparison. To narrow down the data, we took the natural logarithm of each expected loss which can be shown as ln(Exp(A)). The higher the identity attribute owner’s privacy risk score, the higher the property loss they will face when their identity attribute is exposed to danger. To achieve this linear effect, this research finds the highest expected loss in the UTCID ITAP dataset and applies the natural logarithm to it. As a result, this research defines the Privacy Risk score of an identity attribute *A* as

(7) $$score_{risk}\left(A\right)=\frac{ln(Exp(A))}{Max}$$

where Max denotes the maximum value of expected loss after applying natural logarithm. Hence, the Risk score becomes a value that is normalized between 0 and 1.

### 4.4. Scoring for Iot Devices

With the privacy risk score algorithm for an identity attribute, this research computes the privacy risk score for a set of identity attributes collected by IoT devices. Given an IoT device *S* that collects *N* identity attributes, a set of identity attribute can be shown as $ID_{S}={\left\{A_{i}\right\}}_{i=1:N}$. Finally, the total privacy risk score of the collected dataset can be shown as:

(8) $$Score\left(S\right)=\frac{1}{Total}\sum_{i=1}^{N}score_{risk}\left(A_{i}\right)$$

where Total denotes the sum of risk score of the entire UTCID ITAP dataset. Thus, the privacy risk score becomes a value that is also normalized between 0 and 1. Table 3 summarizes the mathematical symbols used in this section.

### 4.5. UTCID Personalized Privacy Assistant for Iot

UTCID Personalized Privacy Assistant for IoT is a mobile application that helps users discover IoT devices and services nearby. Through IRR, our PPA can know which IoT devices and services near the user’s current location may collect the user’s personal information. Our PPA lists the devices registered in the IRR relevant to the user’s current location and the user can see each device’s functionality, ownership, and data practices through the app. If the IoT device or service provides a user option to accept or reject the collection and provision of personal information, this option will also appear in the PPA.

Through the UTCID IoT Identity Ecosystem, users can choose their own identity attributes to generate their own customized Identity Ecosystem Graphic Model. PPA tells users which IoT devices around them are collecting their own data, and can also use the Privacy Risk Score Calculation Algorithm to calculate the IoT device’s impact on their privacy risks through the identity attributes collected by those devices and its own Identity Ecosystem.

**Figure 4:**
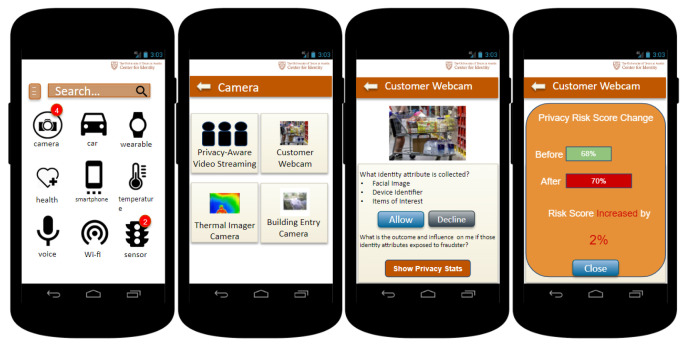
Graphic user interface for UTCID Personalized Privacy Assistant (PPA) for the IoT. The app lists the devices and services available (**left** and **middle-left**) as well as details about the data collection and use practices of a particular resource (**middle-right**), including privacy risk change for the user (**right**).

Figure 4 shows the graphic user interface example of the PPA. A user walks into a store that uses a smart camera system that uses facial recognition and behavioral tracking to determine what items the customers linger around, indicating their potential interest. The system contains a database of known faces and associated contact information. When the system recognizes a customer interested in a particular item, it sends him/her a promotional message. This system and related IoT devices have been registered in the IRR. This enables the owner of the store to notify customers about the use of smart cameras and also allows the owner to expose an opt-in privacy setting for facial recognition, as the camera system is configurable. To decide if he/she wants to allow the data collection of the smart camera system, the user first takes a look at the privacy risk score changes. After considering the 2% increase is a minor change, the user presses the allow button to agree with the smart camera to collect their identity attributes.

## 5. Experimental Results

We show some interesting findings and results from each part of our proposed solution.

### 5.1. Comparison of Target Set to Dataset Collected by Popular Open-Source Apps

**Figure 5:**
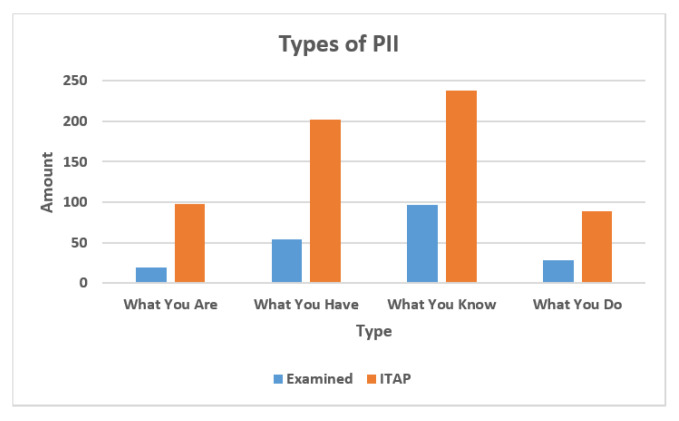
This figure includes the portion of 4 types of PII in examined set and ITAP set.

From our previous work [8], our examination of popular apps for iOS and Android phones showed that around 35% (220 out of 627) of identity attributes in the ITAP list are collected by popular apps. For convenience, this research calls this set the examined set. Figure 5 shows the percentage of each identity attribute type in the examined set and in the ITAP list, respectively. We assume that the ITAP list is representative of all identity attributes. In the examined set, the type of “What You Know” and “What You Have” are most frequently collected by the mobile apps. That is, the mobile applications often want to collect information known privately by a person and information assigned by other organizations. The ITAP list has the same distribution of the number of each type of identity attribute.

Aside from the previous findings, in the examined set of identity attributes, 4% of the ITAP list of identity attributes, are collected by all the popular apps. We show some of these identity attributes in Table 4. These identity attributes such as Name, Address, Date of Birth, and Email Address are often entered by users during the registration process or users just make the attributes visible on their social media. This active exposure of identity attributes is a part of the users’ digital footprint. For convenience, we call this 4% of identity attributes the target set.

The top 10 most compromised internationally identity attributes in 2019 are shown in Table 5 [7]. Such high-risk and valuable identity attributes also appear in the target set. Recall that each identity attribute in the ITAP reference list has several characteristics including risk of exposure and monetary value. We first aggregate the risk of exposure of the target set and compare it with the total risk of exposure of the ITAP reference dataset. The result shows that the risk of exposure of the target set actually accounts for 64.7% of the total aggregate risk of exposure of the ITAP reference attributes. This shows that most of types of identity attributes that popular apps tend to collect belong to high-risk groups. On the other hand, we sum the value of the identity attributes (monetized value of attributes in theft and fraud cases) in the target set and compare it with the total value of the ITAP dataset. The result shows that the value of the target set accounts for 24.8% of the ITAP dataset. Just 4% of the identity attribute in the ITAP dataset accounts for nearly a quarter of the monetary value. Recall that the value of each identity attribute is the average value of monetary losses in more than 6000 incidents collected by ITAP. So this 24.8% sum of cost (about 300 million US dollars) is just an average value, which means the real money loss may be lower or higher.

Now we get back to the target set – identity attributes collected by the popular mobile app. In the previous work [50], we have collected the privacy score of 10 most popular open-source android apps. We show them with the privacy score of the target set in Table 6. Higher privacy scores indicate that an app introduces higher privacy risk to the user.

Take the app “Telegram” for example. This research first goes through its privacy policy, which can be found in its Github repositories or their website, to extract the set of identity attribute that this app tends to collect and share. Second, this research look up the ITAP dataset to find the risk of exposure (e.g., P(A) for identity attribute A) and liability value (e.g., L(A) for identity attribute A) of each identity attribute that “Telegram” collects. Then, this research applies Formulas (1) and (2) to get the accessibility and post effect of each identity attributes that “Telegram” collects. Then, this research applies Formulas (4)–(7) to get the individual privacy risk score for each identity attributes. Finally, to summarize the total privacy risk score, this research applies the Formula (8). Therefore, we are able to get the score of “Telegram” which is 73.99 in Table 6.

The privacy score of the target set is 36.69 which is close to the lowest score in the top 10 open-source apps which is remarkable since that the target set includes only 4% of identity attributes in the ITAP dataset.

Now let us take a look at the data collection in the physical world. Table 7 includes identity attributes required by the N-400 form [51] which we use when applying for Naturalization at U.S. Citizenship and Immigration Services (USCIS). This set of identity attributes has a privacy score of 26.8. This score is lower than the score of target set which is only a small part of identity attributes collected by many apps on our mobile phones.

Another interesting finding is that, for each attribute $A_{k}$, in the UTCID Identity Ecosystem, there is a set of out-degree edges denoted as $E_{out}\left(A_{k}\right)$ and the set of nodes directly connected with $E_{out}\left(A_{k}\right)$ is the set of first level children denoted as $CHILDREN_{first}\left(A_{k}\right)$. When an identity attribute is targeted by a perpetrator, its first level children are the most vulnerable. The edge between the two nodes by definition means that when this identity node is violated by a perpetrator, the next node that may be violated is one of its first level children. The total number of identity attributes collected by the investigated mobile apps (the target set) is about 4% of the ITAP list. In addition the target set and $CHILDREN_{first}\left(A_{k}\right)$ (for $A_{k}$ in the target set) together cover 30% of the entire ITAP list. This indicates that this 4% of identity attributes actually has a significant number of out-degree edges in the UTCID Identity Ecosystem graph model and thereby the exposure of this small 4% can have a significant impact on making other identity attributes more vulnerable to exposure.

### 5.2. Comparison between Personalized Privacy Assistants

The fundamentals and design recommendations of personalized privacy assistant will be discussed in this section. This research also compares the proposed privacy assistant to other existing works.

Colnago et al. [52] use semi-structured interviews with 17 participants to find out what users think about PPA and whether they reject it, so as to determine the user’s attitude towards the benefits and problems of PPA’s autonomy. We list the features and recommendations they derive from those interviews that they think the privacy assistant should obtains.

- **Multiple Recommendations Sources**: Users are aware of potential biases and agendas influencing recommendations from PPA. A good design for this functionality would allow users to pick the types of recommendation sources that they would prefer.
- **Crowd-sourced**: Recommendations based on real users’ opinions and social cues.
- **Authoritative source**: Recommendations based on expert opinions, manufacturers and independent organizations.
- **Trusted Location**: Remove unnecessary notifications by incorporating a “trusted location” feature, such that users would not be notified about devices in those locations.
- **Setting Configuration**: PPA could list devices once and request a decision, revisiting that decision periodically if new devices were added or the user’s preferences changed. Another way to avoid unnecessary notifications is let the PPA learn the user’s preference setting or to specify data collection situations where users are always opposed to or always in favor of sharing.
- **Explanations of risks and benefits**: Users sometimes may feel nothing from privacy-concerning contexts because the risks did not seem to be dangerous to them or could do any harm to them. To mitigate this, recommendations from PPA should offer users detailed and transparent explanations of risks, benefits, and consequences. This can help users understand what the collection and use of these identity attributes have to do with them and why they need to care, and make them more comfortable and decisive when making judgments and decisions.

Table 8 shows the comparison between existing works of PPA and the proposed solution of this research. “Mult-RS” stands for multiple recommendations sources. “Crd-src” stands for crowd-sourced. “Auth-src” stands for authoritative source. “Tru-Loc” stands for trusted location. “Set-Conf” stands for setting configuration. “Exp-RB” stands for explanations of risks and benefits. From this table, we can observe the advantages of our proposed work. All three existing work have the feature “Setting Configuration”. It is done either by saving the setting or type of IoT devices in advance or by using machine learning or deep learning techniques. On the other hand, our proposed solution not only leaverages the UTCID ITAP and Identity Ecosystem, which are authoritative sources, but also shows the outcome and consequences of allowing the data collection from specify IoT devices. Unfortunately, no existing work offers multiple resources of recommendations, crowd-source, or trusted location. To improve our PPA, the future work would be implementing the setting configuration with machine learning technique or finding another resource to calculate the privacy risk score to give users different type of recommendations to follow.

## 6. Conclusions

This research describes innovative approaches for constructing a Personalized Privacy Assistant (PPA) for IoT devices, the range and characterization of identity attributes characterizing IoT devices (eg, serial number, processor type, port numbers, power usage, etc.), the connection of those IoT device identity attributes to identity attributes describing People, and the portion of IoT identity attributes that can best related to and identify People.

This research endeavors to determine the static attributes used to identity IoT devices and those identity attributes dynamically generated from IoT devices in order to gain a comprehensive set of identity attribute, understand how IoT devices can be described and identified and to populate a identity graph model of IoT devices in UTCID Identity Ecosystem. This research built relationships between all identity attribute nodes to form a graph model IoT device identity attributes connected (related) to the identity attributes describing people in the UTCID Identity Ecosystem. The model offers an insight into how personally identifiable information is utilized within the digital world. The research provides a qualitative and quantitative description of the connections, connecting the identity attributes of People, and Devices.

To mitigate privacy risks, this research conducts a framework to estimate the privacy risk score of each IoT device by computing the expected loss the set of identity attributes that each IoT device collects, according to the IoT privacy infrastructure this device registered to. The proposed approach leveraged the identity attributes collected from these devices and cross-referenced these identity attributes to a list of over 600 identity attributes collected in a reference UTCID Identity Ecosystem graph model (where identity attributes were first identified in ITAP threat cases). This research proposed a basic approach with the intrinsic property of identity attributes and an advanced approach with dynamic properties to estimate the privacy risk score of each mobile App. Leveraging two defined properties, Risk and Value, for each identity attribute this research calculates the privacy risk score of each identity attribute. This research also adds two parameters resulting from the UTCID Ecosystem probabilistic models and Bayesian inference tool, Accessibility (discoverability of the identity attribute) and Post Effect (impact in terms of exposing other identity attributes), to refine the original Risk of exposure and Value of monetary loss for each identity attribute.

This research shows the fundamental features of the PPA and compares those features and advantages to other existing work. Unlike other existing work who are giving recommendations based on real users’ opinions, preferences, and social cues, the proposed PPA in this research gives recommendations based on expert opinions, and independent organizations (UTCID ITAP and IoT Identity Ecosystem). Moreover, the proposed PPA in this research offers users tangible explanations of risks and benefits based on the privacy risk score framework, while we have been fairly successful at calculating the IoT privacy risk to the user and helping users better understand and relate to the consequences of IoT data collection and sharing, the IoT presents a significantly broader set of scenarios and contexts. Therefore, the limitation is that the proposed PPA does not support multiple scenarios and another critical issue is that there is no regulated or open-source privacy infrastructure yet. Hence, the IoT PPA is not accessible and not freely downloadable until there is a regulated or open-source IoT privacy infrastructure. When a regulated IoT privacy infrastructure becomes apparent, the issue left is how we can access it with the IoT PPA. Until then, the goal of our IoT PPA is to support multiple scenarios to work in the inevitable upcoming smart city society.

## Figures and Tables

**Table 1:** Summary of the relevant studies for identity attributes and IoT devices.

Work	Category	Advantages	Gap
[12,13,14,15,16,17]	Identity Attributes in General	Analyzation for mobile apps to detect identity attributes shared from or generated by the app.	A comprehensive analysis of identity attribute collection, sharing and abuse. A comprehensive analysis of identity attribute occurrences, dependencies, value and risk across multiple cases of sharing and abuse.
[18,19,20,21,22,23,24,25,26,27,28,29,30,31,32]	Devices and Privacy	Focuses on the detection of actual mobile app data transmissions to third parties or mobile apps accessing data	Does not allow for user’s run-time preventive solutions. Program analysis requires experts, is conducted off-line, and is time-consuming and expensive. Detection of actual data transmission only allows for reactive solutions. Consequences of personal data transmissions to third parties not identified.
[33,34,35,36,37,38,39,40]	Privacy Risk Estimation	Analyzation for privacy policies to assess governance and regulation compliance.	Does not focus privacy risk posed by mobile apps. Lacks calculation of direct privacy risk to users.
[9,11,41,42]	Privacy Assistant	provides users suggestions on their privacy settings based on users’ setting preferences.	Does not suggest settings based on user’s privacy risk. Does not let users know why one identity attribute is more important than another.

**Table 2:** Some examples for identity attributes of devices.

PortNumbers	PowerFrequency	PowerUsage
ProcessorType	Reputation	SerialNo
ApplicationType	BusType	Cache
CircuitDesign	Color	CookieWipe

**Table 3:** A list of mathematical symbols used in this section.

Symbol	Meaning
*V*	The set of identity attributes in UTCID IoT Identity Ecosystem.
*E*	The set of directed edges.
*G*	The graph the UTCID IoT Identity Ecosystem represents as.
P(A)	The probability of exposure for identity attribute *A*.
L(A)	The liability value for identity attribute *A*.
Anc(A)	The set of ancestors for identity attribute *A*.
Des(A)	The set of descendants for identity attribute *A*.
$P^{\prime }{\left(A\right)}_{A_{k}}$	The probability of identity attribute *A* gets exposed on its own after its ancestor $A_{k}$ was exposed.
AC(A)	The accessibility for identity attribute *A*.
PE(A)	The post effect for identity attribute *A*.
Exp(A)	The expected loss for identity attribute *A*.
$score_{risk}\left(A\right)$	The privacy risk score for identity attribute *A*.
Score(S)	The privacy risk score for IoT device *S*.

**Table 4:** Some common identity attributes collected by popular apps.

Password	Username	Name	Login Credentials
Phone Number	Account Information	Email Address	Zip Code
Security Q&A	Time Stamp	Date	URL
User Sign Up Date	Address	Last Login Date	Age
Date of Birth	Gender	IP Address	Location

**Table 5:** Top 10 compromised identity attributes in 2019.

1. Name	2. Address	3. Email Address	4. Phone Number	5. Dat of Birth
6. Credit Card	7. Password	8. Username	9. Bank Account	10. Debit Card

**Table 6:** The comparison of target dataset and popular open-source Apps.

App	Score (%)
Target set	36.69
N-400	26.8
Wiki	43.63
Firefox Focus	47.99
Kodi	48.79
QsmFurthermore,	54.51
Duckduckgo	67.39
OpenVPN	68.92
Signal Private Messenger	69.32
Ted	71.82
Blockchain Wallet	73.67
Telegram	73.99

**Table 7:** List of identity attributes in N-400 form.

Data Set Required at USCIS			
1. MilitaryId	2. Military Service Record	3. Email	4. SSN
5. Phone Number	6. Travel History	7. Fingerprints	8. Parents Name
9. Parents Occupation	10. Spouse Info	11. Address	12. Birth Certificate
13. Hometown	14. School	15. Organization	16. Signature
17. Citizenship	18. Zip Code	19. Name	20. Date of Birth
21. Height	22. Weight	23. Gender	24. Eye Color
25. Hair Color	26. Ethnicity	27. Crime History	28. Age

**Table 8:** Existing works and features of personalized privacy assistant.

Work	Mult-RS	Crd-src	Auth-src	Tru-Loc	Set-Conf	Exp-RB
This research			Yes			Yes
Das et al. [11]					Yes	
Feng et al. [9]					Yes	
Ayci et al. [41]					Yes	

## Data Availability

No new data were created or analyzed in this study. Data sharing is not applicable to this article.

## Abbreviations

The following abbreviations are used in this manuscript:

UTCID	University of Texas at Austin, Center for Identity
ITAP	The Identity Threat Assessment and Prediction
PII	Personally Identifiable Information
PPA	Personal Privacy Assistant
IoT	Internet of Things
OSN	Online Social Networks
DNS	Domain Name System
NLP	Natural Language Processing

## References

[1] Deloitte (2023). Shiny new devices may be bringing joy, but who’s protecting consumer data?. Deloitte Insights.

[2] IBM (2022). Cost of a Data Breach Report 2022.

[3] Landi H. (2021). Fitbit, apple user data exposed in breach impacting 61M fitness tracker records. Fierce Healthc..

[4] University of Texas at Austin About the Center for Identity. https://identity.utexas.edu/about-center-identity..

[5] Zaiss J., Nokhbeh Zaeem R., Barber K.S. (2019). Identity Threat Assessment and Prediction. J. Consum. Aff..

[6] Zaeem R.N., Manoharan M., Yang Y., Barber K.S. (2017). Modeling and analysis of identity threat behaviors through text mining of identity theft stories. Comput. Secur..

[7] Zaiss J., Anderson R., Zaeem R.N., Barber K.S. (2019). ITAP Report 2019. https://identity.utexas.edu/2019-itap-report-0.

[8] Chang K.C., Nokhbeh Zaeem R., Barber K.S. Is Your Phone You? How Privacy Policies of Mobile Apps Allow the Use of Your Personally Identifiable Information. Proceedings of the 2020 Second IEEE International Conference on Trust, Privacy and Security in Intelligent Systems and Applications (TPS-ISA).

[9] Feng Y., Yao Y., Sadeh N. (2021). A Design Space for Privacy Choices: Towards Meaningful Privacy Control in the Internet of Things. Proceedings of the 2021 CHI Conference on Human Factors in Computing Systems.

[10] Carrez F., Elsaleh T., Gómez D., Sánchez L., Lanza J., Grace P. A Reference Architecture for federating IoT infrastructures supporting semantic interoperability. Proceedings of the 2017 European Conference on Networks and Communications (EuCNC).

[11] Das A., Degeling M., Smullen D., Sadeh N. (2018). Personalized privacy assistants for the internet of things: Providing users with notice and choice. IEEE Pervasive Comput..

[12] Krishnamurthy B., Wills C.E. (2009). On the Leakage of Personally Identifiable Information via Online Social Networks. Proceedings of the 2nd ACM Workshop on Online Social Networks.

[13] Humphreys L., Gill P., Krishnamurthy B. (2014). Twitter: A content analysis of personal information. Inf. Commun. Soc..

[14] Aura T., Kuhn T.A., Roe M. (2006). Scanning Electronic Documents for Personally Identifiable Information. Proceedings of the 5th ACM Workshop on Privacy in Electronic Society.

[15] Ranchal R., Bhargava B., Othmane L.B., Lilien L., Kim A., Kang M., Linderman M. Protection of Identity Information in Cloud Computing without Trusted Third Party. Proceedings of the 2010 29th IEEE Symposium on Reliable Distributed Systems.

[16] Weingärtner R., Westphall C.M. A Design Towards Personally Identifiable Information Control and Awareness in OpenID Connect Identity Providers. Proceedings of the 2017 IEEE International Conference on Computer and Information Technology (CIT).

[17] Arefi M.N., Alexander G., Crandall J.R. (2018). PIITracker: Automatic Tracking of Personally Identifiable Information in Windows. Proceedings of the 11th European Workshop on Systems Security.

[18] Canbay Y., Ulker M., Sagiroglu S. Detection of mobile applications leaking sensitive data. Proceedings of the 2017 5th International Symposium on Digital Forensic and Security (ISDFS).

[19] Reardon J., Feal Á., Wijesekera P., On A.E.B., Vallina-Rodriguez N., Egelman S. 50 ways to leak your data: An exploration of apps’ circumvention of the android permissions system. Proceedings of the 28th USENIX Security Symposium (USENIX Security 19).

[20] Liu X., Liu J., Zhu S., Wang W., Zhang X. (2019). Privacy risk analysis and mitigation of analytics libraries in the android ecosystem. IEEE Trans. Mob. Comput..

[21] Alazab M., Alazab M., Shalaginov A., Mesleh A., Awajan A. (2020). Intelligent mobile malware detection using permission requests and API calls. Future Gener. Comput. Syst..

[22] Grundy Q., Chiu K., Held F., Continella A., Bero L., Holz R. (2019). Data sharing practices of medicines related apps and the mobile ecosystem: Traffic, content, and network analysis. BMJ.

[23] Huckvale K., Torous J., Larsen M.E. (2019). Assessment of the data sharing and privacy practices of smartphone apps for depression and smoking cessation. JAMA Netw. Open.

[24] He Y., Yang X., Hu B., Wang W. (2019). Dynamic privacy leakage analysis of Android third-party libraries. J. Inf. Secur. Appl..

[25] Shipp L., Blasco J. (2020). How private is your period?: A systematic analysis of menstrual app privacy policies. Proc. Priv. Enhancing Technol..

[26] Salat L., Davis M., Khan N. (2023). DNS Tunnelling, Exfiltration and Detection over Cloud Environments. Sensors.

[27] de Moraes Rossetto A.G., Sega C., Leithardt V.R.Q. (2022). An Architecture for Managing Data Privacy in Healthcare with Blockchain. Sensors.

[28] Babun L., Celik Z.B., McDaniel P., Uluagac A.S. (2019). Real-time analysis of privacy-(un) aware IoT applications. arXiv.

[29] Sharma K., Gupta B.B. (2019). Towards privacy risk analysis in android applications using machine learning approaches. Int. J.-Serv. Mob. Appl..

[30] Han C., Reyes I., Feal Á., Reardon J., Wijesekera P., Vallina-Rodriguez N., Elazar A., Bamberger K.A., Egelman S. (2020). The price is (not) right: Comparing privacy in free and paid apps. Proc. Priv. Enhancing Technol..

[31] Pereira F., Crocker P., Leithardt V.R. (2022). PADRES: Tool for PrivAcy, Data REgulation and Security. SoftwareX.

[32] Barua A., Al Alamin M.A., Hossain M.S., Hossain E. (2022). Security and Privacy Threats for Bluetooth Low Energy in IoT and Wearable Devices: A Comprehensive Survey. IEEE Open J. Commun. Soc..

[33] Au K., Zhou Y., Huang Z., Gill P., Lie D. Short paper: A look at smartphone permission models. Proceedings of the 1st ACM Workshop on Security and Privacy in Smartphones and Mobile Devices.

[34] Felt A.P., Chin E., Hanna S., Song D., Wagner D. (2011). Android Permissions Demystified. Proceedings of the 18th ACM Conference on Computer and Communications Security.

[35] Zaeem R.N., German R.L., Barber K.S. (2018). PrivacyCheck: Automatic Summarization of Privacy Policies Using Data Mining. ACM Trans. Internet Technol..

[36] Nokhbeh Zaeem R., Barber K.S. (2017). A study of web privacy policies across industries. J. Inf. Priv. Secur..

[37] Zaeem R.N., Barber K.S. (2020). The Effect of the GDPR on Privacy Policies: Recent Progress and Future Promise. ACM Trans. Manag. Inf. Syst..

[38] O’Loughlin K., Neary M., Adkins E.C., Schueller S.M. (2019). Reviewing the data security and privacy policies of mobile apps for depression. Internet Interv..

[39] Harkous H., Fawaz K., Lebret R., Schaub F., Shin K.G., Aberer K. (2018). Polisis: Automated Analysis and Presentation of Privacy Policies Using Deep Learning. Proceedings of the 27th USENIX Security Symposium (USENIX Security 18).

[40] Zuo C., Lin Z., Zhang Y. Why Does Your Data Leak? Uncovering the Data Leakage in Cloud from Mobile Apps. Proceedings of the 2019 IEEE Symposium on Security and Privacy (SP).

[41] Ayci G., Sensoy M., ÖzgÜr A., Yolum P. (2022). A Self-aware Personal Assistant for Making Personalized Privacy Decisions. arXiv.

[42] Stöver A., Hahn S., Kretschmer F., Gerber N. (2023). Investigating How Users Imagine Their Personal Privacy Assistant. Proc. Priv. Enhancing Technol..

[43] Applikey Editorial Team (2018). Most Profitable App Categories; Applikey.

[44] Kuklenko D. (2019). The Most Promising App Categories in 2019. Applikey.

[45] Anthopoulos L.G., Rodríguez-Bolívar M.P. (2015). Understanding the Smart City Domain: A Literature Review. Transforming City Governments for Successful Smart Cities.

[46] Schaffers H., Komninos N., Pallot M., Trousse B., Nilsson M., Oliveira A. (2011). Smart cities and the future internet: Towards cooperation frameworks for open innovation. The Future Internet Assembly.

[47] Gubbi J., Buyya R., Marusic S., Palaniswami M. (2013). Internet of Things (IoT): A vision, architectural elements, and future directions. Future Gener. Comput. Syst..

[48] Zaeem R.N., Manoharan M., Barber K.S. Risk Kit: Highlighting Vulnerable Identity Assets for Specific Age Groups. Proceedings of the 2016 European Intelligence and Security Informatics Conference (EISIC).

[49] Cao Y., Yang L. A survey of Identity Management technology. Proceedings of the 2010 IEEE International Conference on Information Theory and Information Security.

[50] Chang K.C., Zaeem R.N., Barber K.S. (2020). A Framework for Estimating Privacy Risk Scores of Mobile Apps. International Conference on Information Security.

[51] Chang K.C., Zaeem R.N., Barber K.S. Enhancing and evaluating identity privacy and authentication strength by utilizing the identity ecosystem. Proceedings of the 2018 Workshop on Privacy in the Electronic Society.

[52] Colnago J., Feng Y., Palanivel T., Pearman S., Ung M., Acquisti A., Cranor L.F., Sadeh N. (2020). Informing the Design of a Personalized Privacy Assistant for the Internet of Things. Proceedings of the 2020 CHI Conference on Human Factors in Computing Systems.

